# Considerations when measuring myocardial perfusion reserve by cardiovascular magnetic resonance using regadenoson

**DOI:** 10.1186/1532-429X-14-89

**Published:** 2012-12-28

**Authors:** Nicole M Bhave, Benjamin H Freed, Chattanong Yodwut, Denise Kolanczyk, Karin Dill, Roberto M Lang, Victor Mor-Avi, Amit R Patel

**Affiliations:** 1 Departments of Medicine and Radiology, University of Chicago, Cardiac Imaging Center, 5841 S. Maryland Ave., MC5084, Chicago, IL, 60637, USA

**Keywords:** Stress CMR, Regadenoson, Vasodilators

## Abstract

**Background:**

Adenosine cardiovascular magnetic resonance (CMR) can accurately quantify myocardial perfusion reserve. While regadenoson is increasingly employed due to ease of use, imaging protocols have not been standardized. We sought to determine the optimal regadenoson CMR protocol for quantifying myocardial perfusion reserve index (MPRi) – more specifically, whether regadenoson stress imaging should be performed before or after rest imaging.

**Methods:**

Twenty healthy subjects underwent CMR perfusion imaging during resting conditions, during regadenoson-induced hyperemia (0.4 mg), and after 15 min of recovery. In 10/20 subjects, recovery was facilitated with aminophylline (125 mg). Myocardial time-intensity curves were used to obtain left ventricular cavity-normalized myocardial up-slopes. MPRi was calculated in two different ways: as the up-slope ratio of stress to rest (MPRi-rest), and the up-slope ratio of stress to recovery (MPRi-recov).

**Results:**

In all 20 subjects, MPRi-rest was 1.78 ± 0.60. Recovery up-slope did not return to resting levels, regardless of aminophylline use. Among patients not receiving aminophylline, MPRi-recov was 36 ± 16% lower than MPRi-rest (1.13 ± 0.38 vs. 1.82 ± 0.73, *P* = 0.001). In the 10 patients whose recovery was facilitated with aminophylline, MPRi-recov was 20 ± 24% lower than MPRi-rest (1.40 ± 0.35 vs. 1.73 ± 0.43, *P* = 0.04), indicating incomplete reversal. In 3 subjects not receiving aminophylline and 4 subjects receiving aminophylline, up-slope at recovery was greater than at stress, suggesting delayed maximal hyperemia.

**Conclusions:**

MPRi measurements from regadenoson CMR are underestimated if recovery perfusion is used as a substitute for resting perfusion, even when recovery is facilitated with aminophylline. True resting images should be used to allow accurate MPRi quantification. The delayed maximal hyperemia observed in some subjects deserves further study.

**Trial registration:**

ClinicalTrials.gov NCT00871260

## Background

In recent years, vasodilator stress cardiovascular magnetic resonance (CMR) perfusion imaging has been shown to be a sensitive and specific means of diagnosing coronary artery disease [1-9]. CMR also offers a wealth of data regarding myocardial structure and function, without exposing patients to ionizing radiation, and aids in risk stratification for future adverse cardiovascular events [10-12]. Because of these advantages, vasodilator CMR is a rapidly burgeoning methodology.

Most commonly, vasodilator stress CMR is performed with adenosine. This drug requires a continuous infusion, such that separate IV lines are required for vasodilator and contrast agent. Due to activation of A_1_, A_2B_ and A_3_ receptors, adenosine has a variety of undesirable side effects, which include atrioventricular (AV) block, hypotension, and bronchospasm [13]. These occurrences can interrupt workflow and, in rare circumstances, compromise patient safety [14]. Regadenoson, a selective A_2A_ receptor agonist, is an appealing alternative for stress CMR because it is administered as a single, standard-dose bolus (such that only one IV line is required) and has a more favorable side-effect profile [15,16]. We have demonstrated the feasibility and safety of regadenoson CMR and have shown that perfusion defects on stress CMR images predict future need for revascularization [17]. One advantage of stress CMR is the ability to quantify myocardial perfusion reserve (MPR), which is less dependent upon interpreter expertise and improves the accuracy of the detection of multivessel coronary artery disease [18]. Others have shown that MPR obtained by vasodilator stress CMR is similar, regardless of whether hyperemia is induced with adenosine or regadenoson [19].

Because the half-life of adenosine is 2–10 seconds [16], and thus the hyperemic effects of the drug are expected to be completely resolved after 10–15 minutes, stress CMR imaging with adenosine is often performed before rest imaging [10,20,21]. This stress followed by rest approach eliminates the possibility that stress images could be contaminated by delayed enhancement from previously administered gadolinium-based contrast, potentially masking the presence of perfusion defects.

Clinicians often perceive regadenoson as a short-acting agent because its side effects are short-lived, but the drug has a terminal half-life of approximately 2 hours [22]. After administration, it redistributes rapidly throughout the body, followed by slower elimination. Due to regadenoson’s longer half-life, recovery is occasionally facilitated with aminophylline before resting images are acquired [17,23]. However, the residual impact of regadenoson on coronary blood flow during the clearance period is not known.

Accordingly, the goals of this study were: (1) to determine whether a stress-recovery regadenoson CMR protocol can reliably quantify myocardial perfusion reserve as compared to a rest-stress protocol, (2) to ascertain whether post-stress aminophylline administration results in a complete return of myocardial perfusion to a pre-stress level, and (3) to establish a reference range for MPR index (MPRi) in normal volunteers.

## Methods

### Study subjects

Twenty healthy volunteers (70% female, 70% Caucasian, mean age 32 ± 10 yr) were prospectively recruited for vasodilator CMR. Exclusion criteria were: history of coronary artery disease, congestive heart failure, or diabetes mellitus; non-sinus rhythm; any contraindication to regadenoson (including heart block, chronic obstructive pulmonary disease, or severe asthma); and any contraindication to contrast-enhanced CMR (including history of chronic kidney disease with glomerular filtration rate <30 mL/min, implantable pacemaker or defibrillator, severe claustrophobia, or adverse reaction to gadolinium-based contrast agents). All subjects were instructed not to consume caffeine for 12 hours prior to the test. A 12-lead ECG was performed in each subject prior to imaging to rule out high-degree AV nodal block. The University of Chicago institutional review board approved the study.

### CMR image acquisition protocol

#### Myocardial structure and function

CMR images were acquired using a 1.5-T scanner (Achieva, Philips, Best, Netherlands) and a 5-element phased array cardiac coil. Retrospectively gated cine images were obtained with a steady-state free precession (SSFP) sequence (TR 2.9 ms, TE 1.5 ms, flip angle 60°, and temporal resolution ~40 ms). A stack of short-axis slices (8 mm thickness, 2 mm gap) from base to apex was acquired. Standard 2-, 3-, and 4-chamber long-axis views were also obtained.

#### Myocardial perfusion imaging

**Figure 1:**
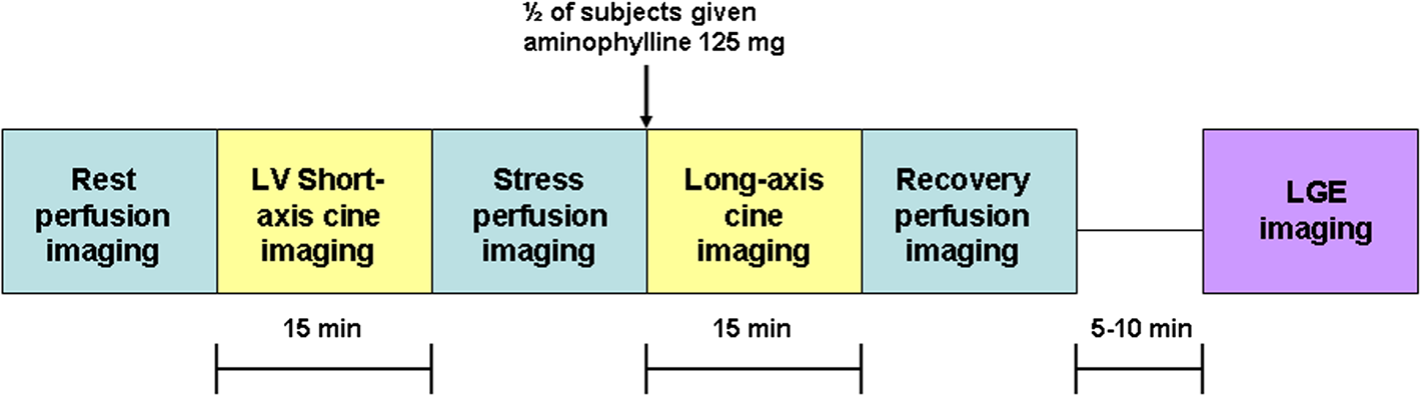
**CMR protocol.** Perfusion imaging sequences are separated by 15-min intervals as shown.

A hybrid gradient echo/echo planar imaging sequence (voxel size ~2.57×2.5 mm, slice thickness 10 mm, flip angle 20°, repetition time 5.9 ms, echo time 2.5 ms, EPI factor 5, delay time 80 ms, and SENSE factor 1.3) was used to acquire short-axis slices at 3 levels of the left ventricle (LV) during first pass of gadobenate dimeglumine (0.05 mmol/kg at 4 mL/sec, 15 patients) or gadolinium-DTPA (0.025 mmol/kg at 4 mL/sec, 5 patients) for 50 consecutive heartbeats. All subjects underwent myocardial perfusion imaging during 3 separate physiologic states, each separated by 15 minutes: (1) resting conditions, (2) 1 minute after regadenoson administration (Lexiscan 0.4 mg IV bolus, Astellas Pharma), and (3) during recovery. In 10 randomly selected subjects, recovery was facilitated with aminophylline (125 mg IV), administered 1 minute after stress imaging (Figure 1). Heart rate was monitored continuously, and mean arterial blood pressure was recorded at rest, at peak stress, and during recovery.

#### Late gadolinium enhancement imaging

Late gadolinium enhancement (LGE) images with slice positions identical to the above described cines were obtained 5–10 minutes after the third infusion of contrast, using a T1-weighted gradient echo pulse sequence with a phase sensitive inversion recovery reconstruction (TR 4.5 ms, TE 2.2 ms, TI 250-300 ms, flip angle 30°, flip angle 5°, voxel size 2x2x10mm, SENSE factor 2). Optimal inversion time was chosen based on a TI scout (typically 250–300 ms).

### Image analysis

**Figure 2:**
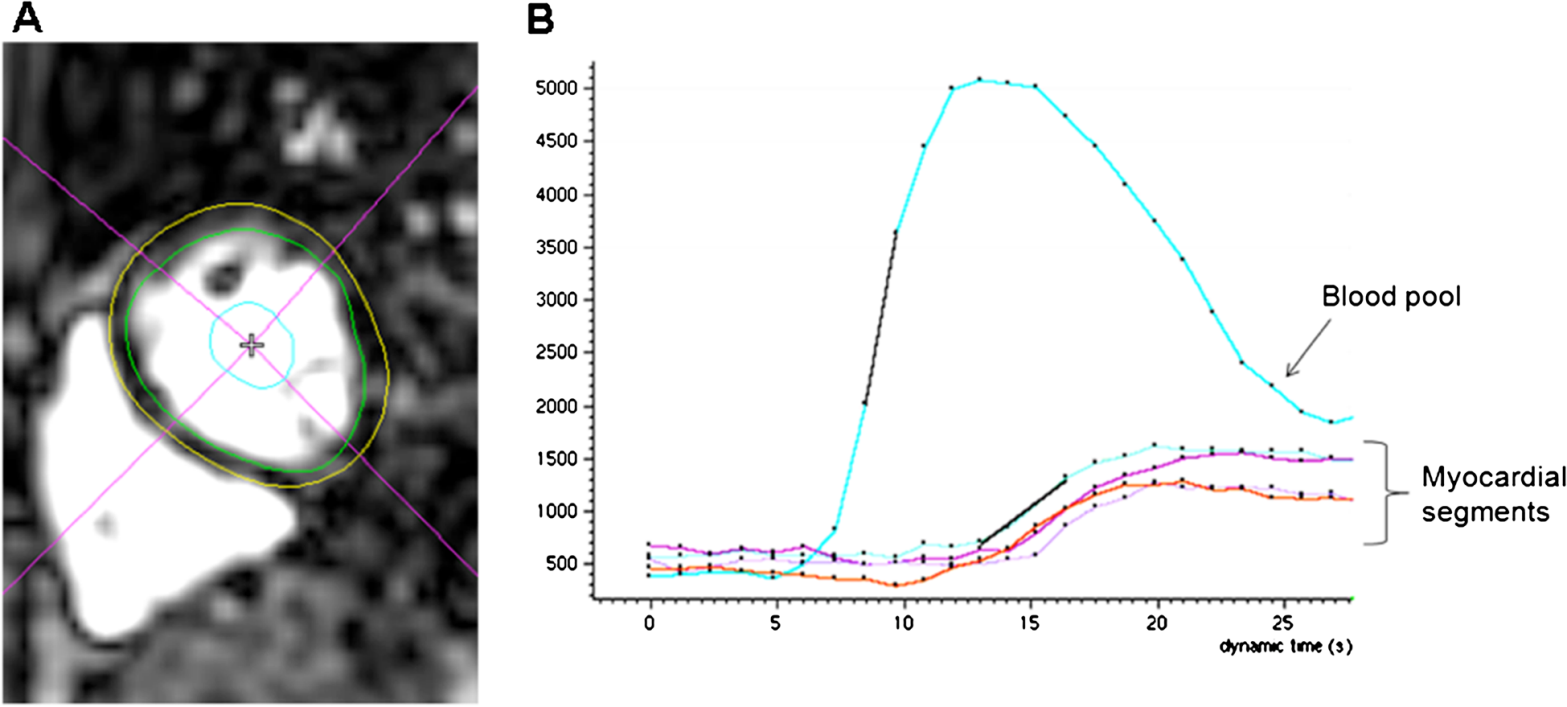
**A. Example of myocardial tracing and segmentation in a short-axis slice.** Endocardial border is shown in green and epicardial border in yellow. The LV myocardium was segmented into 4 sectors (anterior, lateral, inferior, septal). **B.** Blood pool and segmental myocardial time-intensity curves. Maximum up-slopes (black lines) were determined for the blood pool and for each myocardial segment; the mean myocardial up-slope was then normalized to the blood pool up-slope.

Images were analyzed with a commercial software package (Philips View Forum, Best, Netherlands). Short-axis cine slices were used to measure ventricular volumes and ejection fractions (LVEF) using the Simpson method of disks. For semi-quantitative perfusion analysis, epicardial and endocardial borders of the mid-LV slice were manually traced for each frame, myocardial segmentation was applied, and time-signal intensity curves were generated for each myocardial segment and the LV cavity. The maximum myocardial up-slopes were normalized to the LV cavity up-slope and averaged for all myocardial segments (Figure 2) . MPRi was calculated as both (1) the up-slope ratio of stress to rest (MPRi-rest) and (2) the up-slope ratio of stress to recovery (MPRi-recov). Cine, perfusion, and LGE images were visually assessed by a CMR expert (ARP).

### Statistical analysis

Continuous variables were analyzed using student’s t-tests (paired for intragroup comparisons, unpaired for intergroup comparisons; equal variances were not assumed). Categorical variables were analyzed with Fisher’s exact test.

## Results

Table 1 shows the basic characteristics and hemodynamic data of the two groups of subjects: 10 subjects who received aminophylline and the remaining 10 subjects. All subjects had normal LVEFs (>50%) and LV end-diastolic volume indices, with no regional wall motion abnormalities, late gadolinium enhancement, or perfusion defects.

**Table 1:** Patient demographics and hemodynamic responses to regadenoson

	**Aminophylline**	**No aminophylline**	***P *value**
	**(*n*** **= 10)**	**(*n*** **= 10)**	
**Age (yr)**	32 (7)	32 (13)	0.90
**% female**	70	50	0.65
**% Caucasian**	70	50	0.65
**LVEF (%)**	61 (5)	61 (5)	0.97
**LVEDV index (mL/m**^**2**^**)**	85 (9)	84 (14)	0.89
**Baseline HR (BPM)**	68 (9)	65 (14)	0.67
**HR change, baseline to stress (BPM)**	+55 (11)	+48 (13)	0.22
**HR change, stress to recovery (BPM)**	−48 (11)	−35 (12)	0.03
**HR change, rest to recovery (BPM)**	+7 (9)	+13 (12)	0.24
**Baseline MAP (mmHg)**	77 (11)	78 (12)	0.75
**Change in MAP, baseline to stress (mmHg)**	+3 (10)	+4 (7)	0.81

*SD*, standard deviation; *LVEF*, left ventricular ejection fraction; *LVEDV*, left ventricular end-diastolic volume; *HR*, heart rate; *BPM*, beats per minute; *MAP*, mean arterial pressure. Data in parentheses are standard deviations.

In all subjects, regadenoson significantly increased the heart rate above baseline (mean increase 52 ± 13 beats per min, *P* < 0.001). Patients receiving aminophylline post-stress had a significantly larger fall in heart rate from stress to recovery (mean decrease 48 ± 11 vs. 35 ± 12 beats per min, *P* = 0.03; Table 1). Common side effects of regadenoson included dyspnea or difficulty with breath holds (45%), palpitations (35%), chest pressure or heaviness (35%), and flushing (25%). Two subjects reported dysgeusia immediately following aminophylline administration. Side effects did not cause any significant delays in scanning, and no adverse events occurred.

**Figure 3:**
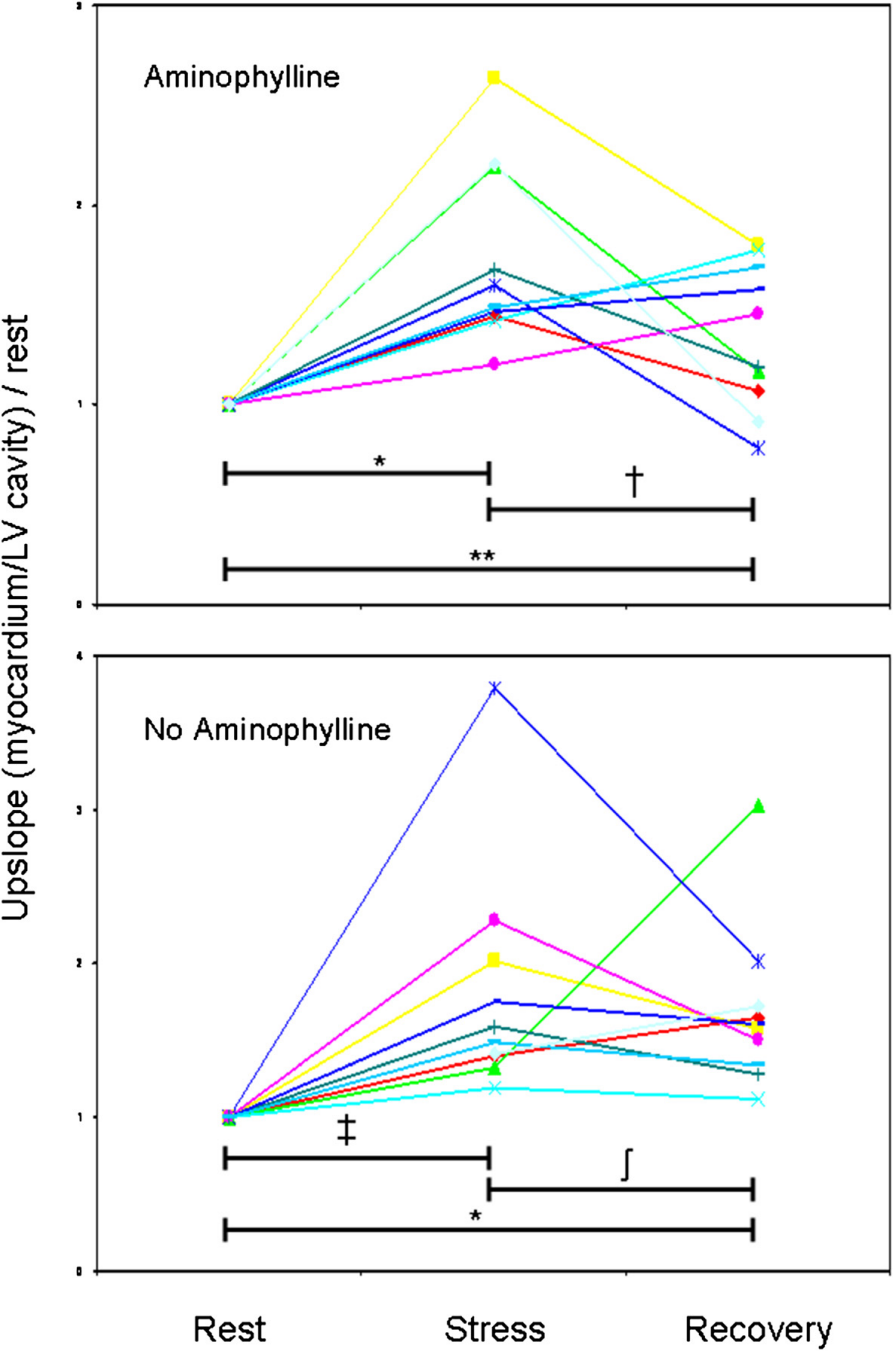
**Comparison of perfusion up-slopes at stress and recovery, normalized for rest up-slope, on a per-patient basis.** Four subjects in the aminophylline group and 3 subjects in the no-aminophylline group had greater perfusion at recovery than at stress. ^*^ P < 0.001; ^†^ P = 0.08; ^**^P = 0.02; ^‡^ P = 0.01; ^ʃ^ P = 0.38

In all 20 subjects, myocardial perfusion (as estimated by normalized up-slope) increased from rest to stress: mean rest up-slope was 8.26 ± 2.40, and stress up-slope was 14.50 ± 6.21 (*P* < 0.001). Regardless of aminophylline administration, recovery up-slope was higher than rest up-slope and not significantly different from stress up-slope (Table 2). Among patients who did not receive aminophylline, myocardial perfusion up-slope was consistently greater at recovery than at rest (mean increase 68 ± 54%, *P* < 0.01; Figure 3). In the aminophylline-facilitated recovery group, all but 2 subjects had greater perfusion up-slopes at recovery than at rest (mean increase 34 ± 37%, *P* = 0.02).

**Table 2:** Regadenoson CMR perfusion up-slopes at rest, stress, and recovery, with and without aminophylline

	**Rest up-slope (SD)**	**Stress up-slope (SD)**	**Recovery up-slope (SD)**	**P value, recovery vs. rest**	**P value, recovery vs. stress**
**No Aminophylline**	8.79 (2.98)	15.99 (8.38)	14.08 (4.03)	<0.001	0.64
**Aminophylline**	7.73 (1.64)	13.01 (2.53)	10.19 (2.98)	0.02	0.08

Data in parentheses are standard deviations.

Mean MPRi-rest for all 20 subjects was 1.78 ± 0.60. Regardless of aminophylline administration, MPRi-recov was lower than MPRi-rest, suggesting incomplete recovery from hyperemia. In the non-aminophylline group, mean MPRi-recov was 36 ± 16% lower than MPRi-rest (1.13 ± 0.38 vs. 1.82 ± 0.73, *P* = 0.001). In the aminophylline group, mean MPRi-recov was 20 ± 24% lower than MPRi-rest (1.40 ± 0.35 vs. 1.73 ± 0.43, *P* = 0.04; Table 3). Although the absolute difference between MPRi-rest and MPRi-recov tended to be smaller in the aminophylline group as compared to the non-aminophylline group, this difference was not statistically significant (*P* = 0.11 for intergroup comparison).

**Table 3:** Comparison of aminophylline and no-aminophylline groups with respect to perfusion reserve

	**MPRi-rest, mean (SD)**	**MPRi-recov, mean (SD)**	**% difference (SD)**	***P *value**
**No Aminophylline**	1.83 (0.73)	1.13 (0.38)	36	0.001
**Aminophylline**	1.73 (0.43)	1.40 (0.35)	20	0.04

*MPRi-rest*, myocardial perfusion reserve index comparing stress to rest; *MPRi-recov*, myocardial perfusion reserve index comparing stress to recovery. Data in parentheses are standard deviations.

Three subjects in the non-aminophylline group and 4 in the aminophylline group had increased myocardial perfusion indices at recovery as compared to stress (Figure 3). These 7 patients exhibited no significant differences in age, body surface area, or gender distribution as compared to all other subjects. Baseline diastolic blood pressure was slightly higher in this group (68 ± 12 vs. 55 ± 11 mmHg, *P* = 0.04). Although subjects in this group tended to have greater heart rate increases at stress, this difference was not statistically significant (56 vs. 50 bpm, *P* = 0.33). Subjects with more hyperemia at recovery than at stress, by definition, all had MPRi-recov < 1, and MPRi-recov was 43 ± 11% lower than MPRi-rest (0.80 ± 0.16 vs. 1.39 ± 0.10, *P* < 0.001). As compared to subjects who were maximally hyperemic at stress, those who were maximally hyperemic at recovery had lower MPRi-rest (1.39 ± 0.10 vs. 1.99 ± 0.68, *P* = 0.008), lower MPRi-recov (0.80 ± 0.16 vs. 1.52 ± 0.41, *P* < 0.001), and higher recovery-to-rest ratios (1.84 ± 0.53 vs. 1.33 ± 0.35, *P* = 0.05). The recovery-to-rest ratios in the delayed hyperemia group were similar to MPRi-rest in the remaining patients (1.84 ± 0.53 vs. 1.99 ± 0.68, *P* = 0.58).

## Discussion

Vasodilator stress CMR is being increasingly incorporated into clinical practice for the evaluation of coronary artery disease because of its superior diagnostic performance when compared to single photon emission computed tomography (SPECT). Because of its ease of use and improved side-effect profile compared to adenosine, regadenoson is being used more frequently to induce hyperemia during vasodilator stress CMR. However, optimal protocols for regadenoson CMR have not yet been established. In this study, we found that: (1) regadenoson-induced hyperemia persists to a degree, even after 15 minutes of recovery; (2) recovery facilitated with aminophylline only partially reverses the residual regadenoson-induced hyperemia; and (3) some individuals may have delayed maximal hyperemia following the administration of regadenoson. Therefore, when regadenoson myocardial perfusion imaging is performed prior to recovery perfusion imaging, the calculated myocardial perfusion reserve may significantly underestimate the actual myocardial perfusion reserve, irrespective of whether aminophylline is used to reverse hyperemia.

One particular strength of stress CMR is its ability to assess myocardial perfusion, quantitatively or semi-quantitatively, as an adjunct to visual analysis. Assessment of myocardial perfusion reserve has been shown to reduce inter-reader variability and to quantify more accurately the severity of localized ischemia due to epicardial coronary stenosis [24], particularly when ischemia is present in multiple vascular territories [18]. Myocardial perfusion reserve by CMR can also reveal diffuse ischemia attributable to microvascular dysfunction [25], and is a potentially useful tool for identifying subclinical disease in patients with cardiovascular risk factors [26,27].

We have established, albeit in a relatively small group of normal subjects, a reference range for MPRi-rest with regadenoson, as determined semi-quantitatively using the time-intensity up-slope technique with a hybrid gradient echo/echo planar imaging sequence. Perfusion analysis by this method is rapid enough for routine clinical use and does not require cumbersome dual-bolus imaging, which may be desirable when MPR is determined quantitatively by deconvolution methods [28]. DiBella and colleagues have previously compared perfusion reserve with regadenoson to that with adenosine and found good agreement, using an ultra-fast gradient echo sequence (Turbo FLASH, Siemens) and deconvolution. However, their study included patients with CAD and cardiovascular risk factors, so their findings cannot be used as reference values [19]. Based on our mean MPRi-rest 1.78 ± 0.60, we calculate that a 58-patient sample size would be needed to detect a 25% difference in perfusion reserve between normals and abnormals (80% power, α = 0.05). Accordingly, in future drug trials aimed at treatment of microvascular dysfunction, regadenoson stress CMR could allow detection of significant differences in perfusion reserve in relatively small cohorts. Our reported normal ranges may also be useful for the development of appropriate semi-quantitative regadenoson stress CMR cut-off values for detecting significant coronary disease and microvascular dysfunction.

Aminophylline is a dissociable complex of theophylline, a methylxanthine adenosine receptor antagonist, and ethylenediamine, which improves solubility. In one small cardiac catheterization study, aminophylline appeared to reverse regadenoson-induced hyperemia to some degree. In subjects receiving aminophylline, coronary blood flow fell below 2-fold of baseline within <1 min, whereas subjects not receiving aminophylline remained above 2-fold of baseline for an additional 7 min. Notably, at the 10-minute mark, neither group of subjects returned to baseline perfusion [23].

In our subjects, we suspect that persistent hyperemia occurred in the post-regadenoson recovery phase due to residual vasodilatory effects of the drug, given its relatively long terminal half-life [23]. Although regadenoson has a relatively weak affinity for the A_2A_ receptor [29], such that its peak vasodilatory effect is brief, drug that is distributed throughout the tissues may return to the blood compartment during recovery and cause a low level of coronary vasodilation. While aminophylline seems to help alleviate the side effects of regadenoson, and is therefore often used as an antidote, its ability to reverse the coronary vasodilatory effects of regadenoson has not been fully described. In our subjects, aminophylline did not eliminate the vasodilatory effects of regadenoson – that is, some degree of hyperemia persisted at recovery in most subjects who received aminophylline. Our results are consistent with those of the previously mentioned catheterization study, in which intracoronary blood flow was measured invasively after regadenoson administration; although the average peak blood flow velocity was reduced by aminophylline, it did not return to baseline by the 10-minute mark [23]. A potential explanation for these observations is that theophylline has a low affinity for the A_2A_ receptor [30], so it may dissociate relatively rapidly, allowing some of the residual regadenoson to rebind post-stress. Interestingly, our aminophylline group had a significantly greater decline in heart rate from stress to recovery, suggesting that aminophylline is somewhat active at the A_2A_ receptor at 15-minute follow up. Regardless, our results suggest that the order of the study protocol is critically important if perfusion reserve is to be determined by regadenoson CMR or, similarly, by any other myocardial perfusion imaging modality.

Although the rest-stress protocol must be used to quantify perfusion reserve accurately, it is unknown if this protocol would lower the sensitivity of regadenoson CMR for detecting ischemic perfusion defects. Administering a lower contrast dose at rest, in a manner similar to rest-stress SPECT imaging, or allowing a prolonged delay between rest and stress imaging, might help mitigate this problem. An alternative strategy would consist of a 2-day protocol with stress imaging performed on the first day and rest imaging performed on a subsequent day. The rest portion could be eliminated if obvious perfusion defects were present on the initial stress images. Of course, such an approach would be costly and time-consuming and would require clinical validation to justify widespread use.

Perhaps our most noteworthy finding is that several patients demonstrated more hyperemia at recovery than at stress. In these subjects, recovery-to-rest ratios (MPRi-recov) were similar to stress-to-rest ratios (MPRi-rest) in the remaining subjects, suggesting that some individuals have a delayed maximal hyperemic response to regadenoson. Aminophylline appears to have little impact on this effect. Although all these subjects had an increase in perfusion from rest to stress, as would be expected, mean MPRi-rest and MPRi-recov were significantly lower than in subjects with rapid hyperemia. Interestingly, the proportions of delayed-hyperemia patients in the aminophylline and non-aminophylline groups were similar, leading us to believe that this phenomenon is unlikely to have biased our overall results. Although patients with delayed hyperemia tended to have slightly higher diastolic blood pressures, no values were pathologically elevated, so this is unlikely to be a clinically useful metric. Further studies will be needed to identify potential predictors of and explanations for the delayed-hyperemic response.

The persistence of hyperemia beyond the immediate post-stress period may raise the question of whether patients with flow-limiting coronary stenoses can safely receive regadenoson without prolonged post-procedural monitoring – i.e., whether such patients might still be “ischemic” when discharged from the CMR center immediately after scan completion. However, it is important to note that regadenoson, like adenosine, does not typically cause true ischemia, but rather a relative lack of hyperemia in segments subtended by diseased vessels. Although it is possible that regadenoson causes a prolonged discrepancy in myocardial blood flow between territories supplied by healthy and diseased arteries, it is unlikely that this results in adverse clinical effects, based on the drug’s excellent safety record in the SPECT literature [16].

### Limitations

This study was conducted in a small number of healthy volunteers. However, this number was sufficient to answer the questions we posed with confidence, as confirmed by statistical analysis. Based on common practice with adenosine CMR, we performed recovery imaging 15 minutes post-stress. It is possible that post-regadenoson recovery imaging either earlier (i.e., 2–3 min after aminophylline administration) or later (20–30 min after stress) would have demonstrated a lesser degree of residual hyperemia. As all of our subjects had normal perfusion, we could not assess whether a rest-stress protocol, as opposed to a stress-recovery protocol, would mask the presence of ischemia due to contrast contamination of stress images. Five individuals who received a very low dose of contrast (0.025 mmol/kg) were included in the analysis; however, their MPRi-rest was not significantly different from the other subjects. Finally, it is unknown whether a relatively modest (approximately 20%) underestimation of perfusion reserve, as seen with MPRi-recov in patients receiving aminophylline, would have clinical significance. Future studies are needed to answer this question.

## Conclusions

We have demonstrated that a regadenoson CMR stress-recovery protocol underestimates perfusion reserve compared to a rest-stress protocol. Therefore, for reliable quantification of myocardial perfusion reserve, the latter approach is preferable. Further research will be needed to determine whether a rest-stress protocol would lower the sensitivity of regadenoson CMR for detecting ischemic perfusion defects, and to establish the length of time between rest and stress imaging that would be necessary to eliminate this issue.

We additionally showed that some subjects have a delayed hyperemic response during the supposed recovery phase; future studies are needed to elucidate the clinical relevance and underlying explanation for this finding.

## Abbreviations

CMR: Cardiac magnetic resonance; MPRi: Myocardial perfusion reserve index; MPRi-rest: Up-slope ratio of stress to rest perfusion; MPRi-recov: Up-slope ratio of stress to recovery perfusion; AV: Atrioventricular; MPR: Myocardial perfusion reserve; SSFP: Steady-state free precession; LV: Left ventricle; LGE: Late gadolinium enhancement; LVEF: Left ventricular ejection fraction; SPECT: Single photon emission computed tomography.

## Competing interests

This study was supported by a grant from Astellas Pharma.

## Authors’ contributions

NB acquired and analyzed the majority of the image datasets, performed all statistical analyses, and drafted the manuscript. BF was involved in study design and image acquisition and analysis. CY was involved in image analysis. DK provided expertise regarding the pharmacokinetics and pharmacodynamics of regadenoson. KD was involved in image acquisition. RL and VM provided assistance with manuscript revision. AP conceived of the study and was involved in image acquisition and analysis and manuscript revision. All authors read and approved the final manuscript.

## Disclosures

Research grant from Astellas Pharma.
